# Comparison of preventive health service provision before and after reorganization of primary care in Turkey: a community-based study

**DOI:** 10.1017/S1463423619000069

**Published:** 2019-07-24

**Authors:** Çiğdem Apaydın Kaya, Mehmet Akman, Pemra Cöbek Ünalan, Serap Çifçili, Arzu Uzuner, Esra Akdeniz

**Affiliations:** 1 Department of Family Medicine, Marmara University Medical School, Istanbul, Turkey; 2 Department of Medical Statistics, Marmara University Faculty of Medicine, Istanbul, Turkey

**Keywords:** child health care, Family Health Center, Health Transformation Program, preventive services, primary health care, reproductive health

## Abstract

**Aim:**

To investigate the changes in the provision of preventive health services in terms of woman and child health after reorganization of the primary health care services.

**Background:**

The primary care system in Turkey has undergone fundamental changes as a part of Health Transformation Program during last decade. But there was no community-based study to evaluate these changes.

**Method:**

This community-based and cross-sectional study was conducted in 2010, just before the reorganization of primary care services and in 2015, five year after the reforms. The 30×7 cluster sampling method was used in Zümrütevler quarter of Maltepe District. The socio-demographic characteristics of the participants, the presence of the physician who can be consulted for any health problem, the presence of smokers at home were questioned. The women aged 18 years or older and gave consent provided information about history of pregnancy and birth, the number of follow-ups during pregnancy, family planning method usage, cervical and breast cancer screening, breastfeeding duration, vaccinations, and prophylactic iron and vitamin D supplementation for their children.

**Findings:**

After the reorganization of primary care, more people stated that they had physicians to whom they could consult for all kinds of health problems (27.8 versus 44.7%; *P*<0.001) and that physician was the primary care physician (30.2 versus 64.7%; *P*<0.001). The reported frequency of at least one smoker at home was decreased after reorganization of primary care (63.6 versus 53.1%; *P*=0.034). There were no significant differences in terms unplanned pregnancy, the use of family planning method, the number of pregnancy follow-ups and the frequency of Pap smears and mammography. There are no significant differences in terms of healthy children follow-ups, vaccination, vitamin D and iron supplementation (*P*>0.05). It was found that the duration of total breastfeeding increased after reorganization of primary care (*P*<0.001).

## Background

The primary care (PC) system in Turkey has undergone fundamental changes during last decades (Health Transformation Program – HTP). In the first decade of the millennium family medicine scheme (FMS) was introduced to the Turkish PC sector. The new PC provision in Turkey had three main dimensions. First was replacing the regional health service delivery model to registered patient list based practice in the PC centers. Second in previous organization of PC services, PC centers were responsible for both individual and community-based preventive services. After introduction of FMS, population-based preventive services were assigned to community healthcare centers. Third, in the previous system physicians without any vocational training were thought to be competent for providing PHC services. After HTP, specialized family physicians with appropriate vocational training aimed to practice in PC, however, since there were not enough trained physicians available, all physicians who wanted to serve in PC were given the title ‘Family Physician’ after a short training period. Then they served as contracted PC physicians in Family Health Centers (FHCs). Within these centers, a population of 1000–4000 people was assigned to each family physician. According to Family Medicine Law in Turkey, a family physician is a physician who is obliged to give PC services to an individual with a comprehensive and continuous manner and without discrimination of age, sex and illness. Family physician also provides required mobile health services and works on a full day basis.

Another major change in PC service delivery was financing healthcare providers’ wages based on capitation and a few performance parameters. With these changes, patients were given the right to choose their physicians while the physicians were given the right to choose their patients. It was ensured that expenses such as rent, electricity, water, repair of the FHCs were financed through the payments under the title ‘current payment’. Moreover, the salary, insurance and severance payment of the employees except medical staff in FHCs have begun to be paid by physicians. In addition, at least one ‘Community Health Center’ was established in each district. The reorganization of PC started in 2004 with a pilot scheme in one province, other provinces were included in time and it was completed in late 2010 with the inclusion of Istanbul.

It is stated that with the HTP there have been mobilization in the PHC services, and also preventive maternal and child health services have been strengthened (Akdağ, 2012). In addition to introduction of FMS, series of ongoing focused programs related to preventive care, were also continued. Some of these are ‘Promotion of Breastfeeding and Baby-Friendly Hospitals’, ‘Turkey Strong as Iron’, ‘Prevention of Vitamin D Deficiency and Improvement of Bone Health among Infants’, ‘Iodine Deficiency Disorders and Iodizing Salt’, ‘Turkey Reproductive Health Program’ and ‘National Tobacco Control Program’ (Akdağ, 2008). It was also reported that the scope of preventive health services expanded with the FMS and the programs for promoting healthy life style were included into preventive health services (immunization, pregnancy and well-child follow-ups, health screenings etc.). In addition, it was stated that ‘the individual who benefits from the service’ should be placed at the center of the service, and in principle, the services were based on the needs, demands and expectations of these individuals (Akdağ, 2012).

Previous studies related to the effects of the HTP usually include health care workers’ and patient’s satisfaction and basic health indicators such as number of well-child care visits and maternal and infant mortality rates (Akıncı *et al*., 2012; Atun *et al*., 2013; Sönmez *et al*., 2013; Arslan *et al*., 2016; Bostan *et al*., 2016). Most of the data used in these studies were composed of data collected by the Ministry of Health and the Turkey Statistical Institute. But not much is known about other preventive services (e.g., vitamin D and iron supplementation, blood pressure measurement, etc.). It was reported that provision of preventive services decreased in general but antenatal care and routine childhood vaccines increased according to cross-sectional data reported by family physicians after HTP (Schafer *et al*., 2016; Akman *et al*., 2017). Since there is no referral chain in Turkey, the patients can apply to any health center. Therefore, patients can also receive preventive health care services from different centers and it is a challenge to differentiate received preventive care from different care levels. Our literature search showed the absence of any community-based study to evaluate the changes in the provision of preventive health services after the introduction of FMS.

The purpose of the present research is to investigate the changes in the provision of preventive health services in terms of women’s and children’s health on a community basis after reorganization of the PC services.

## Methods

1) The present community-based and cross-sectional research was carried out in 2010, just before the reorganization of the PHC services and in 2015, five years after FMS, using the 30×7 cluster sampling method (Henderson *et al*., 1982). The data, for both periods were collected in March in Zümrütevler quarter, located in the middle of the district of Maltepe in Istanbul. This quarter is the most crowded quarter of the district with a population of 54 597 in 2010 and 75 258 in 2015 (Turkey Statistical Institute, 2018). Thirty of 81 streets within the quarter selected randomly by lot, and seven households from each street have been allocated randomly. The decisions on starting from which end of the street and on deciding which apartments to be visited in a building were made with toss of a coin. The data were collected through a questionnaire by interviewing face-to-face with people over 18 years old who opened the door and accepted to participate in the study. If the responding person was male, in addition to the responder, a women older than 18 years from the household was invited to participate the study to collect the reproductive and child health information. The socio-demographic characteristics of participants, the presence of a physician who can be consulted for any kind of health problem, the place where this doctor works, the visit by a medical staff, and the presence of an individual smoking at home were questioned.
2) The women were asked about pregnancy and delivery history, the number of follow-ups during her last pregnancy within the last five years, the use of any family planning method, vaccinations, prophylactic vitamin D and iron supplementation, age of supplementary food onset and breastfeeding duration for children aged two years and older. Also, cervical cancer screening with Pap smear test for women aged 35–69 years and mammography for women aged 40–69 years among the household were questioned.

All data were collected at the end of three consecutive weeks following a 3-h data collection training and a pilot study by the Faculty of Medicine students and researchers. A total of 210 households were targeted for both research periods, while 210 households and 875 people living in these were reached in 2010; the research was completed with 810 people living in 192 households in 2015 due to reasons such as, lack of volunteers and the small number of households on the streets and dead-end streets selected by lot. Each survey lasted ~20–30 min.

## Analysis

The data for both years were compared. The fitness of quantitative variables to normal distribution was tested by Kolmogorov–Smirnov test. For intergroup comparisons, *t* test was used for independent groups fitting normal distribution and Mann–Whitney *U* test for variables not fitting normal distribution. *χ* ^2^ test was used for intergroup comparison of categorical data. Pearson or Spearman correlation tests were used for continuous variables. Statistical analyzes were performed using the SPSS 16.0 package program. The value of *P*<0.05 was taken as statistically significant.

## Findings

According to 2010 data 875 people (52% female (F), 48% male (M)) were living in 210 households; and in 2015 810 people (51% F, 49% M) were living in 192 households. The comparison of the socio-demographic characteristics of the interviewed participants, utilization of PC services and the presence of a physician who can be consulted in any kind of health problem is presented in Table 1. Accordingly, after the FMS, more participants stated that they had a physician to whom they could consult for all kinds of health problems (27.8 versus 44.7%, *P*<0.001) and that physician served in the FHCs (30.2 versus 64.7%, *P*<0.001). In addition, participants reported a decrease in the number of households with at least one smoker after FMS. [*n*=131 (63.6%) versus *n*=102 (53.1%); *P*=0.034].

**Table 1:** Comparison of participant characteristics and utilization of primary care services between 2010 and 2015

	2010 (*n*=210)	2015 (*n*=192)	*P*
Sex			
Male	63 (30.0%)	66 (34.4%)	0.348
Female	147 (70.0%)	126 (65.6%)	
Age (mean±SD)	43±14.43	45±12.55	0.167
Education			
Secondary school and lower	146 (70.2%)	118 (63.1%)	0.135
High school and higher	62 (29.8%)	69 (36.9%)	
	*n*=208	*n*=187	
Presence of a physician to consult for any kind of health problem			
Yes	52 (27.8%)	67 (44.7%)	**<0.001**
No	135 (72.2%)	83 (55.3%)	
	(*n*=187)	(*n*=150)	
The workplace of the physician who can be consulted for any kind of health problem			
Primary Health Care Center/FHC	16 (30.2%)	44 (64.7%)	**<0.001**
Other	37 (69.8%)	24 (35.3%)	
	(*n*=53)	(*n*=68)	
Home visits by a medical staff	37 (18.6%)	25 (14.5%)	0.285
	(*n*=199)	(*n*=156)	

FHC=Family Health Center.

## Data associated with women’s reproductive health

Data on socio-demographic characteristics and reproductive health of women are presented in Table 2. Accordingly, women in reproductive age are better educated in 2015; and there are no significant differences in terms of questioned indicators, such as the number of children, unplanned pregnancy, the use of family planning method, and the number of pregnancy follow-ups, and the frequency of Pap smears and mammography. In both periods, about one-third of the women reported having at least one unplanned pregnancy. More than half of the women who did not use family planning stated that they did not use the method because of reasons other than wanting children (Table 2).

**Table 2:** Comparison of characteristics of women participants and utilization of reproductive health services between 2010 and 2015

	2010	2015	*P*
Age (mean±SD)	32±9.3	32±9.1	0.390
	(*n*=240)	(*n*=230)	
Education			
Illiterate/literate	30 (12.6%)	12 (5.5%)	**0.006**
Primary school	77 (32.4%)	62 (28.3%)	
Secondary school	28 (11.8%)	23 (10.5%)	
High school	68 (28.6%)	65 (%29.7%)	
Higher education	35 (14.7%)	57 (%26.0%)	
	(*n*=238)	(*n*=219)	
Social insurance			
Yes	194 (88.2%)	208 (93.3%)	0.064
No	26 (11.8%)	14 (%6.7%)	
	(*n*=220)	(=223)	
Marital age	21±4.1	20±3.44	0.751
	(*n*=126)	(*n*=114)	
First pregnancy age	22±3.9	22±3.56	0.134
	(*n*=117)	(*n*=112)	
Number of follow-ups during last pregnancy	7±3.6	6±2.92	0.164
	(*n*=57)	(*n*=51)	
Number of children	2±1.3	2±1.24	0.772
	(*n*=109)	(*n*=110)	
Family planning			
Yes	61 (57.5%)	76 (66.1%)	0.191
No	45 (42.5%)	39 (33.9%)	
	(*n*=106)	(*n*=115)	
Family planning method			
Withdrawal	8 (13.1%)	9 (11.9)	0.146
Calendar	2 (3.28%)	5 (6.9)	
Condom	15 (24.6%)	25 (32.9)	
Oral contraceptive	8 (13.1%)	3 (3.95)	
Intrauterine device	20 (32.8%)	18 (23.7)	
Injection	3 (4.91%)	2 (2.63)	
Tubal ligation	5 (8.21%)	13 (17.1)	
Vasectomy	0 (0.0%)	1 (1.32)	
	(*n*=61)	(*n*=76)	
Reasons for not using family planning			
Planning pregnancy	18 (40.0%)	17 (43.6%)	0.532
Fear of side effects	1 (2.2%)	2 (5.13%)	
Not married/sexually active	3 (6.67%)	1 (2.56%)	
Menopause	0 (0%)	2 (5.13%)	
Other (unknown, spouse does not want, etc.)	23 (51.1%)	17 (43.6%)	
	(*n*=45)	(*n*=39)	
Number of pregnancy follow-ups (last pregnancy)			
0	2 (3.6%)	0 (0.0%)	0.392
1–3	9 (16.,1%)	9 (17.6%)	
≥4	45 (80.3%)	42 (82.4%)	
	(*n*=56)	(*n*=51)	
Unplanned pregnancy			
Yes	16 (%7.6%)	36 (38.3%)	0.176
No	42 (72.4%)	58 (61.7%)	
	(*n*=58)	(*n*=94)	
Mammography (40–69 years)			
Yes	38 (48.7%)	37 (43.5%)	0.507
No	40 (51.3%)	48 (56.5%)	
	(*n*=78)	(*n*=85)	
Pap smear (30–65 years)			
Yes	59 (48.8%)	29 (55.8%)	0.398
No	59 (51.2%)	23 (44.2%)	
	(*n*=121)	(*n*=52)	

## Data associated with preventive child health services

Data collected from mothers about children aged one to five years are presented in Table 3. There are no significant differences between two years in terms of healthy children follow-ups, vaccination, vitamin D and iron supplementation, which are pretty frequent for both periods. It was observed that well-child care visits of less than half of the children were done at PC. It was found that the duration of total breastfeeding increased after FMS (*P*<0.001).

**Table 3:** Comparison of well child care services between 2010 and 2015

	2010	2015	*P*
Receiving healthy child follow-up			
Yes	32 (94.1%)	29 (96.7%)	0.630
No	2 (5.9%)	1 (3.3%)	
	*n*=34	*n*=30	
Vaccination			
Complete	34 (94.4%)	32 (94.1%)	0.953
Incomplete/cannot remember	2 (5.6%)	2 (5.9%)	
	*n*=36	*n*=34	
Vitamin D supplement			
Yes	31 (93.9%)	28 (84.8%)	0.230
No	2 (6.1%)	5 (15.2%)	
	(*n*=33)	(*n*=33)	
Iron supplement			
Yes	24 (72.7%)	29 (90.6%)	0.108
No	9 (27.3%)	3 (9.4%)	
	(*n*=33)	(*n*=32)	
Follow-up at primary health center			
Yes	9 (42.9%)	15 (45.5%)	0.851
No	12 (57.1%)	18 (54.5%)	
	(*n*=21)	(*n*=33)	
Onset for supplementary food (mean month**±**SD)	5.39±2.63	7.39±5.99	**0.107**
	Median: 6 (IQR: 4–6)	Median: 6 (IQR: 6–10)	
	(*n*=32)	(*n*=33)	
Breastfeeding duration (mean month**±**SD)	8.81±7.22	19.6±8.51	**<0.001**
(for children of 2–5 years old)	Median: 6 (IQR: 3–14)	Median: 23.5 (IQR: 12–24)	
	(*n*=26)	(*n*=32)	

IQR=interquartile range.

It was reported that blood pressure measurement of children between the ages of 2 and 15 was increased after FMS [*n*=20 (18.3%); *n*=38 (38.4%), respectively; *P*<0.001].

## Discussion

The findings of the present community-based research which compared some preventive health services before and after the introduction of FMS in Turkey are as follows: (1) after the introduction of FMS more participants had a physician to whom they could apply for all kinds of health problems working in PC. (2) The number of households with a smoker decreased. (3) After the introduction of FMS, the total duration of breastfeeding increased significantly. (4) Blood pressure measurement among children between the ages of 2 and 15 was increased after the reorganization of the PHC. (5) In both periods, about half of the women had a screening test for cervical cancer and mammography, and there was no significant difference between two periods. (6) There is no difference between women in two periods in terms of using the family planning method, and about one-third of women had unplanned pregnancies in both periods. More than half of the women who did not use the family planning method had a cause other than wanting children. (7) ~80% of the pregnancies were followed at least four times in both periods. (8) Routine well-child visits for children aged one to five years, vaccination, vitamin D and iron supplement rates were similar in both periods, yet less than half of the children in both years were reported to receive well-child care visits in PC.

Although the referral chain has never been conducted in Turkey, increase in the presence of a physician who can be consulted for any kind of health problem is remarkable. What is more, this physician is mentioned to be the PC physician approximately two times more by the participants in 2015. Our findings are in concordance with a previous study comparing PC services in 1993 and 2012 in 28 European countries including Turkey which reported that utilization of PC as first contact of care has been increased but there was a decline in overall preventive services after health care reforms in Turkey (Schafer *et al*., 2016). Regarding maternal and child health, an increase in antenatal care and routine childhood vaccines was reported in 2012 in Turkey compared to 1993, and this increase was associated with performance criterion applied as wage cuts when the given target is not met (Akman *et al*., 2017). However, our results showed no significant difference between 2010 and 2015 in terms of vaccination rates and number of antenatal care visits.

Another positive result of the current research is the decrease of the percentage of households with a smoker (63.6 versus 53.1%). According to the results of the Global Adult Tobacco Survey, it was reported that there was a decrease in the frequency of smoking over the years, and tobacco use over age of 15 was 31.2% in 2008 and 23.2% in 2012. The same rate was 27.3% in 2014 (Republic of Turkey Ministry of Health, 2017). Within the scope of the ‘National Tobacco Control Program’ initiated in 2007 in Turkey, many attempts were made for tobacco control between 2010 and 2015: prohibition of tobacco use in public transportation vehicles, placement of illustrated health warnings on cigarette packs, operation of the ALO 171 Smoking Cessation Counseling Hotline, free delivery of 360 thousand boxes of smoking cessation medicine, tax increase etc. (TAÇESE, 2018). Observed reduction in smoking status may be a result of all these initiatives. However, existence of smoker in every other house suggests that smoking is still a major problem.

Approximately half of all women at recommended age for cancer screening had cervical Pap smear test and mammography at least once in both periods. Cervical cancer and breast cancer screening were not routinely performed in PC during the periods of the present research. However later, the Ministry of Health ensured that these screenings could be carried out at FHCs. According to 2013 data of Turkey Demographic and Health Survey (TDHS-2013), which has been conducted every five years since 1968 and has a sample of strong representation, the average number of children was 2.3 and pregnancy rate was 23.7% among women (Hacettepe University Institute of Population Studies, 2014). The average number of children per woman is about 2 for both years in which this research was conducted and about one-third of women had an unplanned pregnancy at any time of their lives. Nearly half of the women who did not use the family planning method reported that they had reasons other than ‘being sexually inactive, menopausal or wanting children’. This result suggests that there are other factors affecting the decision of using a family planning method. Considering that Turkey’s National Strategic Action Plan on sexual and reproductive health includes preventing unplanned pregnancies and family planning practices among priority response areas, more studies should be conducted on sexual and reproductive health and family physicians and nurses should be reinforced on the issue (General Directorate of Mother and Child Health and Family Planning, 2010). Since reproductive rights are considered as a subcomponent of human rights, it is necessary to ensure that individuals are informed not only in health settings but also in other settings, and that they have free access to reproductive health services (World Health Organization, 2017).

Breastfeeding has been reported to have many benefits for both maternal and infant health (Victora *et al*., 2016). The World Health Organization recommends that every baby should receive breastfeeding alone during the first six months period, supplementary food should be introduced at the sixth month, and breastfeeding should continue until the second year (World Health Organization, 2017). The duration of breastfeeding may vary between societies depending on social and cultural factors. The detection of this variation between different cultures is very important in terms of community health. Initiatives promoting breastfeeding in Turkey were initiated before FMS and continued after, by the Ministry of Health. Istanbul received ‘golden baby friendly province’ title in 2013. As of the end of 2014, more than 50% of family medicine units in Istanbul were baby friendly. In agreement with these improvements, the present research found that the duration of breastfeeding increased between 2010 and 2015, and the most important factor affecting this period was the introduction of FMS. This increase in the duration of breastfeeding may have resulted from the cumulative effect of numerous initiatives listed above. In addition, increased contact with the PC, which is another finding of the present research, may be a contributing factor to the increased duration of breastfeeding. According to the latest TDHS 2013 data, the duration of breastfeeding median in Turkey is 16.7 months.

This number is 23.5 according to the present research conducted in Istanbul in 2015. The discrepancy between studies might be due to the age of child assessed. In the TDHS, the duration of breastfeeding was assessed for the youngest child under three years of age, whereas in our study, the assessment was made for the youngest child between the ages of 2 and 5. The TDHS, which is carried out every five years, reported an increase in the duration of breastfeeding over the years (Hacettepe University Institute of Population Studies, 2014). Data of studies on breastfeeding other than the TDHS are scarce. Existing studies were in general conducted on the mothers who applied to the hospitals and in most of them breastfeeding in the first 12 months was explored. In a study conducted in Istanbul between 2011 and 2012, among mothers who applied to a hospital pediatric polyclinic for well-child care visit, the total duration of breastfeeding for the first two years was reported as 16.4±7.7 months (Bülbül *et al*., 2012). The present research found a reverse relationship between the duration of breastfeeding and the duration of the mother’s education. Similarly, according to TDHS-2013 data, the duration of breastfeeding for mothers with a shorter education period is longer than more educated mothers and male babies are breastfed longer (Yalçın *et al*., 2011; Hacettepe University Institute of Population Studies, 2014). Although this suggests a possible gender discrimination among infants, further study is required for clarification. According to the findings of the study conducted by Bülbül *et al*. (2012), there was a reverse relationship between the duration of breastfeeding and the duration of mother’s education, but there was no difference in terms of the gender of the baby and the number of siblings. Balcı *et al*. reported that breastfeeding duration of more than 6 months increased 1.021 fold as the maternal age increased one year. Also similarly to our study, they reported mothers with lower education levels breastfed their child for longer period (Balcı *et al*., 2012). The employment status of women was not analyzed as a variable in the present research. However, it is known that the increase in the duration of education increased the chance of employment among women. In this case, it is a fact that making arrangements at work to facilitate breastfeeding will increase the duration of breastfeeding for working mothers.

Since April 2004, ‘Turkey Strong as Iron’ program including prophylactic iron supplementation for infants aged 4–12 months and iron treatment for infants with anemia aged 4–24 months has been implemented (Akdağ, 2008); vitamin D is being provided for free and Turkey Reproductive Health Program is being carried out. The findings of the present research show that these national programs were very successful before and after continued to reach its targets after the introduction of FMS. According to the TDHS data, the percentage and frequency of receiving antenatal care has increased over the years, and according to 2013 data, the percentage of having four or more antenatal care visit per pregnant woman was 88.9%. This figure is higher in the urban area. These figures are similar to the ones obtained in the present research, and the percentages of having four or more antenatal care visit per pregnant woman for both years were lower than the average of Turkey (80.3 and 82.4%).

One of the main health indicators reflecting the condition of the health care system is the utilization of the PC centers. According to the findings of the present research, infant vaccination rates, prophylactic vitamin D and iron supplementation rates were significantly higher in both periods, although the frequency of admissions to FHCs was not analyzed. This finding suggests for both years well-child care visits were held in PC. Although the use of these services was very high for both periods and there was no difference between them, it is interesting to note that less than half of the children were reported to be followed up in PC. This finding suggests that understood by the mothers from PC follow-ups is not only vaccination and prophylactic medicine administration to children, but also follow-ups by the physicians, because, in FHCs, application of vaccines and giving some prophylactic medicines to children are usually performed by a medical staff except physician. However, this issue was not elaborated in the present research.

In a previous study on the factors affecting the working conditions of physicians after the reorganization of the PC services, most of the physicians reported to have increased workload (Ak, 2013). Indeed, although the number of physicians working in the PC increased by 11% from 2002 to 2010, the number of patients treated in the PC increased by 2.5 times (Republic of Turkey Ministry of Health, 2012). The frequency of referral to PC was 3.1 in 2011, while it was 1.1 in 2002 (Ministry of Health 2011 statistics yearbook). The high number of patients enrolled in PC and high frequency of referral may be an obstacle for preventive healthcare provision.

The strength of the present research is that it is community-based. The limitations are the sample is representative for regions of urban areas like Zümrütevler, but does not represent Istanbul or Turkey. So the results may not be generalizable to diverse populations. There is some missing data due to unanswered questions like reproductive health questions. However, we believe that the findings are important because the number of studies comparing before and after the reorganization of PC in Turkey is limited.

In conclusion, the findings of the present study suggest that preventive child health services such as vaccination and prophylactic drug supplementation used to be carried out before the reorganization of PC, are carried out equally well and breastfeeding duration increased. In addition, our study suggests that attempts should be performed to improve the reproductive health services for women.
